# Removal of scleral buckle: indications, long-term outcomes and comparison with the literature

**DOI:** 10.1186/s40942-026-00839-w

**Published:** 2026-05-06

**Authors:** Eunice Linh You, Sihame Doukkali, Mélanie Hébert, Mohammadhossein Ghasempourabadi, Kelvin You, David Jin, Serge Bourgault, Mathieu Caissie, Éric Tourville, Ali Dirani

**Affiliations:** 1 Department of Ophthalmology, Hôpital du Saint-Sacrement, CHU de Québec – Université Laval, Quebec City, Canada; 2 Department of Ophthalmology, Hôpital du Saint-Sacrement, 1050 Ste-Foy Street, Quebec City, Quebec, G1S 4L8 Canada

**Keywords:** Scleral buckle removal, Recurrent retinal detachment, Infection, Anatomic success, Visual acuity

## Abstract

**Objective:**

To assess the clinical indications for scleral buckle removal (SBR) and evaluate the functional and anatomic outcomes, including the risk of recurrent retinal detachment (RD) following SBR.

**Design:**

Retrospective chart review.

**Methods:**

A single-center analysis of patients operated for SBR was conducted at the Centre hospitalier universitaire de Québec – Université Laval in Quebec, Canada between 2008 and 2023 with a minimum of 1 year follow-up. Data were gathered on preoperative characteristics, indication for SBR, time to SBR, surgical techniques used and postoperative outcomes including final best-corrected visual acuity (BCVA). The primary outcome was the incidence of recurrent RD after SBR.

**Results:**

Among 2375 eyes that had placement of scleral buckle for RD, 35 (1.5%) required SBR. Infection (34%) and pain (31%) were the most common reasons for SBR. The median time from buckle placement to removal was significantly shorter for infectious cases (2.4 months) compared to non-infectious cases (12.6 months) (*p* = 0.006). Four patients (11%) experienced recurrent RD, with 3/4 of those cases occurring when buckle explantation was performed within the first month. Postoperative BCVA at final follow-up improved from logMAR 0.70 to logMAR 0.30 (Snellen equivalent of 20/100 to 20/40). A multivariate logistic regression analysis demonstrated no statistically significant predictors of recurrent RD.

**Conclusion:**

Infection and pain are the leading indications for SBR, with infections requiring earlier removal. Recurrent RD occurred in 11% of cases, especially with early removal, with all recurrences occurring within 3 months of SBR. Despite these risks, visual outcomes post-SBR are generally favorable. Close monitoring during the early postoperative period is therefore recommended.

## Introduction

The placement of a scleral buckle (SB) is a common surgical procedure in the management of rhegmatogenous retinal detachment (RD). It can be used as a standalone technique or combined with pars plana vitrectomy (PPV) [1, 2]. Occasionally, scleral buckle removal (SBR) may be required, occurring in 1% to 24% of cases [3–6].

Indications for SBR are diverse and include exposure, migration, infection, chronic pain, foreign body sensation, granuloma, diplopia, induced myopia and even optic nerve impingement [2–5]. SB infection is one of the most common reasons for removal, with regional variations in causative organisms [7, 8]. Untreated infections may lead to severe complications including panophthalmitis and loss of the eye.

The most concerning complication following SBR is the risk of recurrent RD, reported in up to 34% of cases and typically occurring within the first three months of removal [5, 8, 9]. Therefore, the risk-benefit ratio of SBR must be carefully considered in each case [5].

In this study, we aim to investigate the clinical indications for SBR. We also aim to assess the long-term functional and anatomic outcomes, including rate of recurrent RD and prognosis following SBR.

## Materials & methods

### Study design and population

This study is a retrospective, single-center analysis of all patients previously operated with combined SB and PPV at the Centre hospitalier universitaire de Québec – Université Laval between January 2008 and December 2023 with a minimum follow-up period of 1 year postoperatively. Relevant cases were identified using the Procedural Code for Scleral Buckle Removal. The study complied with the principles outlined in the Declaration of Helsinki and received approval from the Research Ethics Board of the CHU de Québec – Université Laval (Approval Number 2022–5980). Individual consent was waived due to the retrospective design and anonymous nature of the data collection. No financial support was required to conduct this study.

Patients were included if they were over 18 years of age at the time of initial SB placement and underwent an SBR during the specified study period. The decision to proceed with SBR was left at the discretion of the treating surgeon based on individual patient circumstances.

Comprehensive preoperative, intraoperative, and postoperative data were gathered from patient records, including baseline evaluations, intraoperative findings, and follow-up visits.

- *Preoperative Data*: Collected variables included patient age, sex, laterality of the affected eye, previous ocular history, lens status (phakic, pseudophakic, or aphakic), macula status in the initial retinal detachment (classified as “macula-on” or “macula-off”), indication for SBR, presence of PVR, presence of inferior pathology and time from SB placement to SBR.
- *Intraoperative Data*: Collected intraoperative variables included fixation technique (sutures vs. tunnels), type of tamponade agent used (SF_6_, C_3_F_8_, or silicone oil), and any additional intraoperative findings.
- *Postoperative Data*: Outcomes assessed postoperatively encompassed the incidence of recurrent RD, the microbiological organisms isolated, the best-corrected visual acuity (BCVA) pre- and post- SBR, the final retina status (categorized as “retina-on” or “retina-off”), subsequent ocular surgeries, as well as the resolution of initial complaints leading to the SBR. The BCVA represented the best visual acuity recorded in the clinic using the patient’s latest refractive correction and pinhole correction, if applicable.

The primary objectives of this study are to evaluate the clinical indications for SBR and to assess the rate of recurrent RD postoperatively. The secondary objectives of this study include identifying risk factors that increase the risk of having a recurrent RD and worse visual outcomes following SBR.

In addition, a review of other reported cases of SBR in the literature was assessed via a systematic search of EMBASE (1947 to present). The search utilized medical subject headings (MeSH) and keywords related to SBR, with relevant terms expanded iteratively until all pertinent terms were captured. Duplicates were removed using EndNote (v8.2), and initial title and abstract screening was conducted using web-based application Rayyan. The search was last updated on December 2025.

### Statistical analysis

Descriptive characteristics of the sample are presented as median values with interquartile ranges (IQR) [Q_1_, Q_3_] for continuous variables, due to their skewed distribution, which was confirmed by the Shapiro-Wilk test. Categorical variables are summarized as frequencies and percentages. Comparisons across different indications for SBR were made using chi-square tests for categorical variables and the Mann-Whitney U test or Kruskal-Wallis test for continuous variables, as appropriate. A Wilcoxon signed-rank test was conducted to compare preoperative and postoperative outcomes for non-normally distributed data. Visual acuity was treated as a continuous variable, expressed in the logarithm of the minimum angle of resolution (logMAR) scale.

A Kaplan-Meier survival analysis was conducted to assess the time to SBR across different removal indications. The survival curves for each indication category were generated, and the time from SB implantation to SBR was plotted along the x-axis, with the probability of remaining without SBR on the y-axis. Log-rank tests were conducted to determine if there were statistically significant differences in time to removal between the different indications for SBR. Hazard ratios were also calculated where appropriate to quantify the relative risk of buckle removal associated with each indication category.

Median survival times (the time at which 50% of patients had their buckle removed) and 95% confidence intervals were estimated for each category. Additionally, for each group, the 25th and 75th percentile survival times were reported to describe the variability in buckle retention times. All statistical analyses were performed using IBM SPSS Statistics for Mac (version 29.0.2; IBM Corp., Armonk, NY), with statistical significance defined as α = 0.05.

## Results

### Patient baseline and perioperative characteristics

A total of *n* = 2375 eyes underwent combined pars plana vitrectomy (PPV)-SB for RD during the study period. Among these, *n* = 35 (1.5%) eyes underwent SBR.

Patients with SBR had a median [IQR] age of 62 [53, 70] years. Most patients were male (*n* = 24, 69%) and pseudophakic at the time of surgery (*n* = 25, 71%). At the time of initial RD presentation, 19 patients (55%) were classified as having either “macula-split” or “macula-off” status, while 16 patients (45%) were classified as “macula-on.” The tamponade agents used during primary surgery included air (*n* = 7; 20%), SF_6_ (*n* = 12; 34.3%), C_3_F_8_ (*n* = 13; 37.1%) and silicone oil (*n* = 3; 8.6%). SB fixation technique involved horizontal mattress sutures in 29 cases (83%) and scleral tunnels in 6 cases (17%). All patients underwent surgery using an encircling type 41 band, and in ten cases this was supplemented with an additional 3084 segmental element. Average preoperative BCVA was 0.68 [0.30–1.80] logMAR, which corresponds to a Snellen equivalent of 20/100.

The leading reasons for SBR included infection (*n* = 12, 34%), pain (*n* = 11, 31%), strabismus (*n* = 3, 8.6%) and exposure without infection (*n* = 5, 14%). Pain was defined as intolerance to the buckle that served as the primary reason for removal, while cases with objective findings such as extrusion were categorized separately, even if pain was reported. The pain was typically neuropathic, often severe, and refractory to standard analgesics, neuropathic agents such as gabapentin, and supportive measures like lubricant drops without significant objective findings that can be easily treatable including exposed sutures, erosion or ocular surface irritation. All three strabismus cases involved restrictive, vertical strabismus with either esotropia (*n* = 1) or exotropia (*n* = 2). Other causes (*n* = 4, 11.4%) included recurrent granuloma formation, increased IOP and/or chronic inflammation. Baseline and perioperative characteristics stratified by reason for SBR are presented in Table 1.

**Table 1:** Baseline characteristics and demographics of patients stratified by cause for removal of scleral buckle

Characteristic, *n* (%), median (IQR)	Infection *n* = 12	Intolerance *n* = 11	Extrusion *n* = 5	Others *n* = 4	Diplopia *n* = 3	*p*-value
**Age**, ***years***	68 (60–75)	61 (38–65)	68 (65–81)	54 (31–62)	57 (44–57)	**0.049**
**Affected eye**, ***left***	4 (33%)	5 (46%)	0 (0%)	3 (75%)	1 (33%)	0.212
**Sex**, ***male***	9 (75%)	3 (27%)	5 (100%)	4 (100%)	3 (100%)	**0.006**
**Lens status**						
Phakic	7 (58%)	10 (91%)	4 (80%)	2 (50%)	2 (67%)	0.159
Pseudophakic	5 (42%)	1 (9%)	0 (0%)	2 (50%)	1 (33%)	
Aphakic	0 (0%)	0 (0%)	1 (20%)	0 (0%)	0 (0%)	
**Macula status**						
On	4 (33%)	5 (45%)	4 (80%)	3 (75%)	0 (0%)	0.135
Off	8 (67%)	6 (55%)	1 (20%)	1 (25%)	3 (100%)	
**Tamponade agent**						
Air	2 (17%)	2 (18%)	2 (40%)	1 (25%)	0 (0%)	0.135
SF_6_	6 (50%)	5 (46%)	0 (0%)	1 (25%)	0 (0%)	
C_3_F_8_	4 (33%)	4 (36%)	1 (20%)	2 (50%)	2 (67%)	
Silicone oil	0 (0%)	0 (0%)	2 (40%)	0 (0%)	1 (33%)	
**SB technique**						
Sutures	3 (25%)	0 (0%)	1 (20%)	1 (25%)	0 (0%)	0.411
Scleral tunnel	9 (75%)	11 (100%)	4 (80%)	3 (75%)	3 (100%)	

Only sex and age distributions indicated some differences across the different reasons for SBR (*p* < 0.05). Post-hoc pairwise analyses indicated that patients with SBR due to infection were older than those who underwent SBR due to diplopia (*p* = 0.018), pain (*p* = 0.051) and other reasons (*p* = 0.058). Female patients were also more likely to have SBR for pain than for extrusion (*p* = 0.007), infection (*p* = 0.022), diplopia (*p* = 0.024) and other reasons (*p* = 0.013). None of these analyses met the criteria for significance after applying the Bonferroni correction for multiple comparisons (adjusted threshold *p* < 0.005).

### Follow-up and outcomes

The average follow-up for all patients was 58.8 [26.61 to 114.81] months. The average time from SB placement to removal was 7.43 [2.1–22.7] months. Postoperative outcomes of patients stratified by cause for removal of scleral buckle are presented in Table 2.

**Table 2:** Postoperative outcomes of patients stratified by cause for removal of scleral buckle

Characteristic, *n* (%), median (IQR)	Infection *n* = 12	Intolerance *n* = 11	Extrusion *n* = 5	Others *n* = 4	Diplopia *n* = 3	*p*-value
**SB survival**, ***months***	2.4 (1.2–8.2)	20.1 (6.6–26.1)	50.7 (5.4–86.2)	4.3 (1.8–18.4)	9.7 (6.3–9.7)	**0.034**
**Recurrence of RD**	3 (25%)	2 (18%)	1 (20%)	0 (0%)	0 (0%)	0.734
**Time to recurrence of RD**, ***months***	31 (14–31.5)	68.5 (24–113)	N/A	N/A	N/A	0.304
**BCVA**, ***logMAR***						
Pre-op SBR	0.87 (0.59–2.4)	0.37 (0.18–0.91)	0.6 (0.09–1.2)	0.79 (0.40–2.18)	2.9 (0.22–2.9)	0.306
Post-op SBR	0.38 (0.24–1.63	0.34 (0.04–0.84)	0.80 (0.075–2.1)	0.14 (0.045–0.70)	0.84 (0.040–0.84)	0.570
Change in BCVA	0.34 (-0.65-1.015)	0.080 (0.017–0.28)	-0.2 (-0.85–0.015)	0.44 (0.090–1.945)	2.06 (0.18–2.06)	0.609
**Evisceration**	1 (8.3%)	1 (9.1%)	0 (0%)	0 (0%)	0 (0%)	0.892
**Total follow-up time**, ***months***	42.7 (15.2-117.4)	108 (81.1-126.4)	51.4 (20.7–80.9)	33.9 (14.9–81.4)	27.1 (26.3–36.3)	0.400

**Fig. 1:**
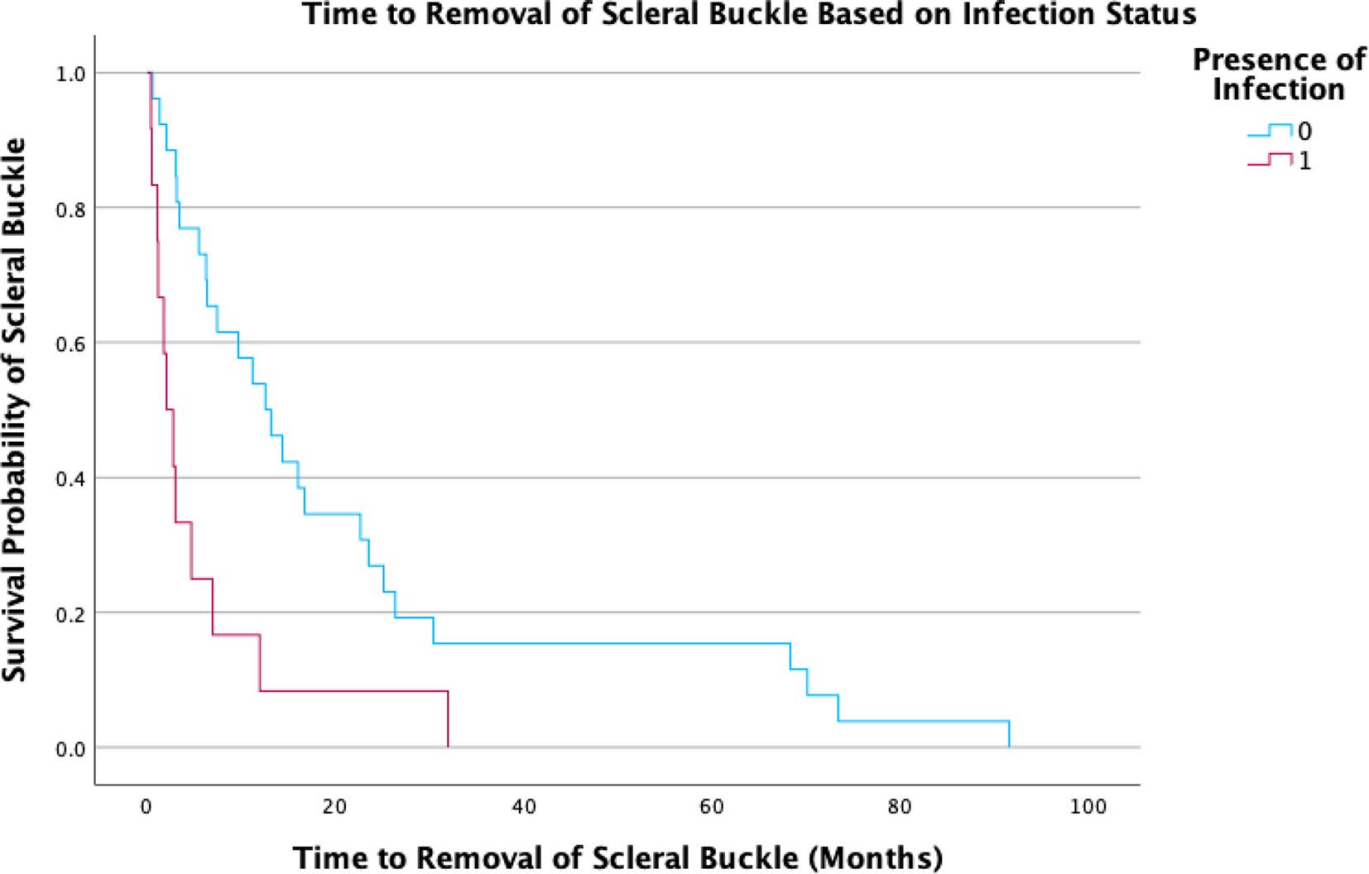
Kaplan Meier survival curve showing the time to removal of scleral buckle based on infection status

Patients with SBR for infectious causes had a significantly earlier time to buckle removal compared to non-infectious causes (2.4 [1.2–8.2] months vs. 12.6 [5.5-25.13] months) (*p* = 0.006). The Kaplan Meier curve is presented in Fig. 1. SBR resolved the initial complaint in all but one case in which there was persistent neuropathic pain. Average postoperative BCVA after SBR improved to 0.36 [0.10–0.99], corresponding to a Snellen equivalent of 20/40. There were no significant differences in outcomes based on reason for SBR.

Four eyes (11%) developed recurrent RD post SBR, with 3 of the cases occurring in patients who underwent SBR within the first month (median delay of 1.3 [0.77–36.8] months). The cause of the early removal was infection in 2 cases and severe pain in 1 case. Two of the 4 cases had documented PVR or vitreous traction prior to SBR. All 4 RD recurrences occurred within the first three months of SBR (median 2.6, [1.3, 3.8] months). In 2 of the patients there were new breaks and progression of PVR. One case had inferior tractional component. All cases were managed using a standard surgical approach including vitrectomy with a combination of heavy perfluorocarbon liquid, endolaser photocoagulation, cryotherapy, and retinectomy.

Two patients had a successful repair. The other two patients developed inoperable RD that ended in evisceration. The first case involved a patient with multiple surgeries for recurrent inferior retinal detachment complicated by macular proliferative vitreoretinopathy. Six years later, the patient opted for SBR, followed by silicone oil removal, for persistent neuralgia despite understanding the associated risks. The second case involved a patient with a presumed SB infection that rapidly progressed to orbital cellulitis and panophthalmitis, requiring urgent SBR. Both patients ultimately developed inoperable recurrent RD with severe PVR, requiring evisceration for a blind painful eye.

All 12 patients with infectious SBR were treated with either systemic oral or intravenous antibiotics and topical antibiotics, including fortified antibiotics in 3 patients. Additionally, all patients received subconjunctival antibiotics perioperatively, and 1 patient received a tap and inject. Cultures were sent for 10 patients, which came out positive for *Staphylococcus sp.* in all cases, including for the previously described case of orbital cellulitis/ panophthalmitis.

In a multivariable Firth‑penalized logistic regression, presence of PVR showed the strongest association with recurrent retinal detachment (OR 4.1, 95% CI 0.5–57.9, *p* = 0.15), followed by inferior retinal pathology (OR 3.7, 95% CI 0.3–75.6, *p* = 0.23 ) and male sex (OR 3.4, 95% CI 0.21–44.8, *p* = 0.32). None of these associations reached statistical significance **(**Table 3**).**

**Table 3:** Multiple logistic regression model for risk of recurrent RD after SBR based on baseline and perioperative characteristics

Characteristic	OR	95% CI	*p*-value
Male	3.4	0.21, 44.8	0.32
Age	1.02	0.94, 1.13	0.50
Infection	2.6	0.28, 31.5	0.37
Duration of SB, *months*	1.03	0.90, 1.17	0.56
Fixation technique	1.8	0.17, 22.1	0.65
Presence of proliferative PVR	4.1	0.5. 57.9	0.15
Inferior pathology	3.7	0.3–75.6	0.23
Tamponade agent	0.42	0.03–6.59	0.48

CI = confidence interval; PVR = proliferative vitreoretinopathy; SB = scleral buckle

## Discussion

This study aimed to identify the indications for SBR and evaluate outcomes, including the rate of recurrent RD following SBR. We found that infection and pain were the most common reasons for SBR, accounting for 34% and 31% of cases, respectively. These findings are consistent with previous reports indicating that infection and mechanical complications are among the most common reasons for SBR [7, 8, 10, 11]. Infections leading to SBR occurred significantly earlier than non-infectious causes, as evidenced by a shorter time to buckle removal for infectious cases (median 2.4 months vs. 12.6 months, *p* = 0.006). Older patients were also more likely to undergo SBR for infection compared to other causes, although this relationship also did not reach the threshold for statistical significance after correction. This is expected as infectious SB require quicker treatment to prevent unfavorable outcomes including progression to panophthalmitis and even loss of the eye. Patients with infections may also experience worse outcomes due to the complications arising from the infection, rather than from the removal of the SB.

We assessed the specific microbiological organisms in 10 of 12 infectious SBR cases and identified *Staphylococcus sp* in all samples. This finding aligns with other studies report *Staphylococcus sp* as the most common isolate, presumably from the patient’s skin flora. Gram-negative, acid-fast organisms and polymicrobial infections have also been documented [7, 11, 12].

The second most common indication for SBR was pain. The analysis of sex and age differences revealed that female patients were more likely to have SBR for pain compared to other indications such as infection, extrusion, and diplopia. In 1977, Schwartz et al. reported pain as primary reason for SB removal in only 5% of patients compared to more recent studies by Le Rouic et al., 2003, Deokule et al., 2003 and Nuzzi et al., 2008 who noted pain as the main indication in 16.5%, 40% and 70% of patients respectively, with an apparent increasing trend of pain being a primary indication of removal over the decades [13–15]. Meanwhile, other studies did not specifically list pain as an indication, although report it in up to 88% of patients in the cohort [5].

The third most common cause was exposure. Although some studies use the term extrusion synonymously with infection, we chose to distinguish extrusion from acute signs of infection. SB extrusion is a very common indication for removal, accounting for over half of cases in recent studies [5, 9, 16–18]. Subclinical erosion may also be under-recognized, with studies reporting extrusion in up to 21.7% of postmortem eyes.[19].

Exposure or extrusion of a SB does not always require surgical removal, with some cases being observed for over a decade without deterioration [20]. The choice to remove a SB should be tailored to individual circumstances, considering factors such as patient age, existing health conditions, how symptomatic the exposed SB is and the visual status of the fellow eye [3].

The overall rate of recurrent RD following SBR in our cohort was 11%, representing 4 of 35 patients, which is consistent to modern rates reported in the literature of up to 12% **(**Table 4**).** An older report by Ulbrich and Burton from 1974 reported a rate of 34% that is widely quoted but this study only included infected buckles and most were removed within 3 months, so a higher rate of redetachment would be expected. In a recent study from our group, the rate of recurrent RD was 11% in patients who underwent PPV-SB, with a surgical success rate of 75% following a second surgery. Therefore, the rate of recurrent RD after SBR appears to be comparable to the expected primary success rate, regardless of SB removal [21]. Furthermore, it is worth noting that patients who are offered a SB in addition to the vitrectomy are also likely to have more complex initial pathology such as severe PVR, inferior lesions, multiple or complex breaks, or advanced detachment which may independently influence recurrence risk and should be considered before attributing redetachment solely to buckle removal.

Additionally, only three patients underwent removal of the buckle within the first month of placement and all three patients had a recurrent RD, signifying the high risk of RD if removed early before the retina has had a chance to properly attach. Early removals were driven by urgent clinical indications such as infection (*n* = 2) or severe pain (*n* = 1). Recurrent RD often also occurred in the early postoperative period, within the first three months of removal for all 4 cases (median 2.6 [IQR 1.3, 3.8] months). We noted a half of SBR occurred within the first 6 months of placement, while the other half occurred to up to over a decade later. This has similarly been noted by Moisseiev et al., 2017 in which a quarter of patients were operated within 6 months and the other quarter were operated after 10 years. The bimodal distribution may represent two distinct causes, with earlier cases occurring due to infection from improperly covered conjunctiva and sharp edges causing extrusion to later causes from long-term erosion of the conjunctiva [16].

On logistic regression analysis, no factors reached statistical significance due to the limited number of cases. However, there was a trend towards higher risk of recurrent RD in patients with presence of PVR, inferior pathology and male patients. These findings are consistent with prior studies showing that eyes with PVR or inferior retinal breaks are more prone to recurrent detachment due to persistent tractional forces, subretinal fluid trapping, and surgical access challenges [22, 23]. The reason for the increased risk of recurrent RD in males is not clear but has been supported by other studies [24–26]. It is thought that biological factors such as abnormal adhesions in the vitreoretinal interface and longer axial lengths seen in males may drive these increased rates of recurrence [24–26]. Other risk factors reported in the literature for redetachment include vitreous traction [27, 28], shorter duration [29], retinal tears (as opposed to holes) and unrecognized retinal breaks [3].

Finally, the mean postoperative visual outcomes improved on average from logMAR 0.7 (Snellen equivalent of 20/100) to logMAR 0.3 (Snellen equivalent 20/40) at final follow-up (*p* = 0.02, Wilcoxon signed-rank test), likely reflecting proper refractive correction in the postoperative period.

SBR was also considered successful in addressing the primary presenting complaint in most patients. All infectious cases demonstrated clinical resolution following removal and antibiotic therapy. There was only one case in which the pain persisted despite the removal of the buckle. Restrictive strabismus and diplopia symptoms also improved in the 3 patients after buckle removal and subsequent muscle surgeries/prism therapy. Although the risk of serious complications including inoperable RD resulting in the loss of the eye in 2 of 35 patients cannot be overlooked, our results suggest that removing the buckle for symptoms such as pain, infection and strabismus effectively alleviates the presenting issues without necessarily compromising visual outcomes for most patients. This encouraging finding is consistent with other reports in the literature which showed SBR was effective for symptom relief as well as clearance of infection [5, 9].

### Limitations

This study has several limitations. First, the retrospective nature of the analysis introduces the potential for selection bias and missing data. We only included cases of combined PPV-SB cases in the analyses as standalone SB surgeries are performed infrequently at our center. Furthermore, there is likely be a selection bias in which only patients with combined procedures were offered SBR compared to patients with SB alone. These results may therefore not necessarily be applicable to eyes with standalone SB, as eyes that underwent combined PPV with internal retinopexy and tamponade may behave differently from eyes with standalone SB. The study was conducted at a single center, which may limit the generalizability of our findings to other populations with different clinical practices and patient demographics. Additionally, the relatively small sample size of patients undergoing SBR may limit the statistical power to detect associations, especially in subgroup analyses. Further studies with larger sample sizes are needed to confirm this finding [24–26].

Another limitation is the lack of standardized criteria for deciding when to perform SBR. The decision was left to the discretion of individual surgeons, which could lead to variability in timing and indications for SBR. This variability may affect outcomes and complicates the interpretation of factors associated with recurrent RD.

Lastly, microbiological results were not available for all cases of SBR, and cases of extrusion without acute signs of infection were not cultured, which limits our ability to rule out subclinical infections. Future studies should aim to include more detailed microbiological data to better understand the relationship between infection and risk of complications.

A summary table of other case series in the literature are included **(**Table 4**).**

**Table 4:** Review of case series on SBR in the literature

Author, year	Sample size	Indications for SBR	Recurrent RD
Hilton et al., 1978	23 eyes	Infection (*n* = 7, 30%), foreign-body sensation (*n* = 7, 30%), recurrent subconjunctival hemorrhages (*n* = 4, 17%), impingement on the optic nerve (*n* = 1, 4%), and distortion of the macula (*n* = 4, 17%)	*n* = 1 (4%)
Deutsch et al., 1992	61 eyes	Extrusion (*n* = 45, 74%), Infection (*n* = 12, 20%), Diplopia (*n* = 2, 4%), Scleritis (*n* = 2, 4%)	*n* = 5 (8.2%)
Deuokule et al., 2003	72 patients	Extrusion (*n* = 34, 47.2%), Pain (*n* = 29, 40.2%)	*n* = 6 (8.3%)
Le Rouic et al., 2003	90 eyes	Diplopia (*n* = 7, 7.7%), swelling of the buckle (*n* = 34, 38%), extrusion (*n* = 44, 89%), granuloma (*n* = 5, 5.5%)	*n* = 8 (8.8%)
Covert et al., 2008 / Han et al., 2013 (same cohort)	36 patients	Exposure without infection (*n* = 16, 44%), Infection without exposure (*n* = 6, 17%), Infection with exposure (*n* = 6, 17%), Irritation (*n* = 6, 17%), Glaucoma (*n* = 1, 3%), and inhibition of the growth of the eye (*n* = 1, 3%).	*n* = 4 (12%)
Nuzzi et al., 2008	43 eyes	Pain (*n* = 30, 70%), Extrusion (*n* = 17, 40%), Conjunctivitis (*n* = 6, 13%), Diplopia (*n* = 4, 9%)	*n* = 0 (0%)
Rasouli et al., 2014	87 eyes	Extrusion (*n* = 66, 76%), diplopia (8%, *n* = 7), infection (6%, *n* = 5), a combination of extrusion and infection (6%, *n* = 5), and Others (5%, *n* = 4).	*n* = 3 (3.4%)
Kazi et al., 2015	102 eyes	Exposure with infection (*n* = 81, 79.4%), Exposure without infection (*n* = 11, 10.8%), Intraocular infection (*n* = 3, 2.9%), Anterior migration without exposure 3 (2.9), Ahmed valve placement 2 (1.9), Anterior migration with buckle exposure (*n* = 1, 0.98%), Limitation of extraocular muscles function (*n* = 1, 0.98%)	*n* = 7 (6.9%)
Moisseiev et al., 2017	49 eyes	Buckle extrusion (*n* = 28, 57.1%), Infection (*n* = 4, 8.2%), Both (*n* = 1, 26.5%), Strabismus/Diplopia (*n* = 4, 8.2%)	*n* = 4 (8.2%)
Kim et al., 2020	40 eyes	Exposure without infection (*n* = 23, 57.5%), Exposure with infection (*n* = 7, 17.5%), Elevated IOP (*n* = 6, 15%), Strabismus/Diplopia (*n* = 3, 7.5%), Migration of buckle material (*n* = 1, 2.5%)	*n* = 4 (10%)
Eshraghi et al., 2021	50 eyes	Exposure (*n* = 27, 54%), Infection (*n* = 13, 26%), Diplopia (*n* = 8, 16%), Recurrent RD (*n* = 2, 4%)	*n* = 6 (12%)
Patel et al., 2024	86 patients	Exposure (*n* = 53, 61.63%), Infection (*n* = 18, 20.93%), Diplopia/Strabismus (*n* = 17, 19.77%), Migration (*n* = 13, 15.12%), Pain (*n* = 11, 12.79%), Chronic redetachment (*n* = 3, 3.49%), Ptosis (*n* = 3, 3.49%)	*n* = 4 (6.56%)
Our study, 2025	35 patients	Infection (*n* = 12, 34%), Pain (*n* = 11, 31%), Extrusion (*n* = 5, 14%), Diplopia/Strabismus (*n* = 3, 9%), Others (*n* = 4, 11%)	*n* = 4 (11%)

## Conclusion

In conclusion, this study highlights infection and pain as the leading indications for scleral buckle removal, with infection cases requiring removal significantly earlier than non-infectious cases. Recurrent RD is a risk following SBR that often occurs in cases that were explanted within the first month postoperatively. However, the overall rate of recurrent RD (11%) is low and comparable to the reported risk of primary RD recurrence after PPV-SB, even without undergoing a SBR [21]. Unless urgent indication such as infection is present, waiting for removal beyond the critical postoperative period is ideal. Among the measures available to the surgeon to gain time are the use of oral antibiotics to reduce inflammation and pain, as well as removal of only the most obviously infected suture when feasible. These approaches may help postpone scleral buckle removal (SBR) by several weeks or, in some cases, months. Despite these risks, visual outcomes and improvement of the initial clinical indication driving SBR are generally favorable, with most patients experiencing stable or even improved vision at final postoperative visit compared to the preoperative visit to the SBR.

Future research should aim to validate these findings in larger, multicenter cohorts and explore additional predictors of recurrent RD following SBR.

## Data Availability

The data is available upon request.

## Abbreviations

BCVA: Best corrected visual acuity
RD: Retinal detachment
SB: Scleral buckle
SBR: Scleral buckle removal

## Declarations

During the preparation of this work, the authors did not use any generative artificial intelligence tools or services. All content was written, reviewed, and edited solely by the authors, who take full responsibility for the content of the publication.
